# The mitochondrial genome, paternal age and telomere length in humans

**DOI:** 10.1098/rstb.2017.0210

**Published:** 2018-01-15

**Authors:** Abraham Aviv

**Affiliations:** The Center of Human Development and Aging, Rutgers New Jersey Medical School, The State University of New Jersey, Newark, NJ 07103 USA

**Keywords:** telomeres, father, offspring, sperm, mitochondria, evolution

## Abstract

Telomere length (TL) in humans is highly heritable and undergoes progressive age-dependent shortening in somatic cells. By contrast, sperm donated by older men display comparatively long telomeres, presumably because in the male germline, telomeres become longer with age. This puzzling phenomenon might explain why TL in the offspring correlates positively with paternal age. The present communication proposes that mitochondrial DNA polymorphisms and heteroplasmy cause variation in the production of reactive oxygen species, which, in turn, mediate age-dependent selection of germ stem cells with long telomeres and hence sperm with long telomeres. These long telomeres are then inherited by the offspring. The effect of paternal age on the offspring TL might be an evolutionarily driven mechanism that helps regulate TL across the human population.

This article is part of the theme issue ‘Understanding diversity in telomere dynamics’.

## 1. Introduction

In humans, stem cells in the male germline undergo many more replications than those in the female germline [1]. This is the reason that the paternal lineage in humans and perhaps other primates is the main source of *de novo* germline mutations in the offspring [2–4]. The numerous replications of stem cells in the human male germline might also explain the recently discovered phenomenon of the paternal-age-at-conception (PAC) effect on telomere length (TL).

While somatic stem cells experience age-dependent telomere shortening, sperm of older males show comparatively longer telomeres [5–7], which are apparently inherited by the offspring [8,9]. Thus, offspring conceived by older fathers have longer telomeres than their peers [5,10–12]. The PAC effect on TL has also been observed in chimps [13], suggesting that its yet unknown evolutionary role might be of significance in hominids.

Such findings highlight another curious observation related to the mode of inheritance of TL in humans. Although the telomere literature provides conflicting results on the mode of TL inheritance [14–16], convincing findings indicate that TL is more strongly affected through the maternal than paternal lineage [17,18]. Thus, offspring TL is influenced through distinct channels of maternal (greater inheritance) and paternal (PAC) effects; and these effects are already evident in newborns [17]. Notably, the mode of inheritance and parental age effect differ in various species. For instance, TL is heritable in the free-ranging sand lizard and shows a PAC effect on TL in male offspring [19]. In birds, TL is also inherited [20–23], principally through the maternal lineage [21–23], and may show a maternal-age-at-conception effect on the offspring TL [23].

## 2. The mitochondrial genome and telomere length dynamics in the human male and female germlines

Findings showing that newborn TL is influenced more through the maternal than paternal lineage [17] suggest that the mitochondrial genome may play a role in TL dynamics during embryonic/fetal development. This is because the mitochondria are inherited solely from the mother.

The DNA of the human mitochondria, the main source of endogenous reactive oxygen species (ROS), is highly polymorphic, presumably because of evolution-mediated adaptation to different environmental and geographical settings [24]. Mitochondrial DNA (mtDNA) is not only polymorphic but also heteroplasmic, i.e. two or more mtDNA alleles might coexist in different proportions in different cells of the same lineage [24]. Thus, mtDNA polymorphisms and heteroplasmy may, respectively, engender variation in ROS production across individuals and across cells of the same lineage within the individual. As ROS augment replication-dependent TL shortening [25,26], cells that produce lower amounts of ROS should experience less replication-dependent TL shortening.

Owing to the uniparental (asexual) mode of mitochondrial inheritance, the outcomes of mitochondrial mutations cannot be attenuated through germline recombination. However, ‘purifying selection' of mitochondria occurs in primordial stem cells of the female germline, presumably to cull deleterious mutations in the mitochondrial genome, which displays a higher mutational rate than the nuclear genome and increased susceptibility to mutational drift [27,28]. However, little is known about mitochondrial selection dynamics, if any, in the male germline.

Importantly, mitochondria in the male germline are not transmitted to the offspring. Still, in the light of the numerous replications of male germ cells [1], variation in the production of ROS due to underlying mtDNA polymorphism and heteroplasmy might differentially influence TL in sperm, which would be transmitted to offspring. Moreover, while ‘purifying selection' in the female germline principally occurs during embryonic development, in theory, germ stem cell selection in the male germline might occur during extra-uterine life throughout the male's long reproductive period.

## 3. Linking reactive oxygen species production with telomere lengthening in the male germline

Given the difficulties in obtaining human oocytes for research and reliance on TL measurements in a single oocyte (compared with millions of sperm) at a time, information about TL dynamics in the human female germline is scant. That said, there is good consensus that age-dependent telomere lengthening does not occur in the female germline [29]. How then can we explain age-dependent telomere lengthening in the human male germline?

Telomerase, the reverse transcriptase that offsets replication-mediated telomere shortening [30], is largely silent in somatic cells of humans and other long-lived large mammals [31]. By contrast, telomerase activity is robust in the testes and presumably germ stem cells (but not mature spermatozoa) of males in humans [32] and other mammals [33,34]. Given that the male germline undergoes numerous replications during extra-uterine life, it makes intuitive sense that telomerase would minimize or eliminate replication-mediated telomere shortening, thereby avoiding critically short telomeres in the germline, which might compromise fitness. The longer telomeres in sperm of older men suggest, however, that telomerase ‘overshoots', adding telomere repeats to the ends of the chromosomes over and above those lost due to the replication of male germ stem cells [7].

But, there might be another potential explanation for telomere elongation in sperm, namely, age-dependent survival, i.e. selection, of male germ stem cells that produce lower amounts of ROS. These selected germ stem cells would display less TL shortening per replication. If telomerase in the male germline adds a constant amount of telomere repeats with each germ cell replication, age-dependent enrichment of germ stem cells that produce a lower amount of ROS would result in progressively longer telomeres in the germline of older males. In fact, indirect evidence, derived from research in twins, suggests a PAC-mediated preferential selection of germ stem cells with longer telomeres in the male germline [35]. Moreover, as the mitochondria in the male germline are not transmitted to the offspring, mitochondrial variants that produce more ROS would not be selected out across generations of males. Thus, the PAC effect would repeat itself generation after generation.

## 4. Conclusion

TL is a complex genetic trait whose dynamics during the individual's life course (including intra-uterine growth and development) might be influenced by the parental nuclear genetics and maternal (mitochondrial) genetics. This communication synthesizes evidence from diverse disciplines to explain the puzzling findings of long telomeres in sperm of older men and hence the PAC effect on the offspring TL. Whatever the underlying mechanisms of the PAC effect on the offspring TL might be, such a phenomenon adds another dimension of ‘plasticity' to trans-generational TL dynamics. We view the PAC effect as a process whereby older fathers endow their offspring with longer telomeres, but another perspective is that younger fathers endow their offspring with shorter telomeres. In this way, in addition to a host of inherent and environmental factors, paternal age in humans might be a unique mechanism through which TL is regulated across generations.

While TL dynamics in the growing fetus/newborn might be influenced by polymorphisms in the maternal mtDNA, in the end, the PAC effect on the offspring TL might largely (and ironically) reflect variation in the mitochondrial genome that is derived from the offspring's paternal grandmother. The collective influences stemming from the mitochondrial genomes of the paternal grandmother (through PAC) and the mother on the offspring's TL suggest that human TL might be more female-driven than male-driven.

Finally, the proposed model of mitochondria-based stem cell selection in the male germline is founded on the premise that the PAC effect on the offspring TL reflects underlying mechanisms that produce long telomeres in sperm of older versus younger males. However, no study has examined sperm TL dynamics based on longitudinal evaluation of sperm donated by the same men as they get older. Analysis based on longitudinal evaluation of the relationship between sperm TL dynamics and mtDNA polymorphisms and heteroplasmy might thus provide the answer about whether the PAC effect on the offspring TL is driven by mitochondrial genetics in the male germline.

## Data accessibility

This article has no additional data.
